# Editorial: Beneficial microbes and the interconnection between crop mineral nutrition and induced systemic resistance, volume II

**DOI:** 10.3389/fpls.2023.1157296

**Published:** 2023-02-24

**Authors:** Carlos Lucena, Ricardo Aroca, Jianfei Wang, Sabine Dagmar Zimmermann

**Affiliations:** ^1^ Departamento de Agronomía (DAUCO-María de Maeztu Unit of Excellence), Campus de Rabanales CeiA3, Universidad de Córdoba, Córdoba, Spain; ^2^ Departamento de Microbiología del Suelo y Sistemas Simbióticos, Estación Experimental del Zaidín (CSIC), Granada, Spain; ^3^ Anhui University of Science and Technology, Huainan, China; ^4^ IPSiM, Univ Montpellier, CNRS, INRAE, Institut Agro, Montpellier, France

**Keywords:** nutrient deficiency, ISR eliciting microbes, crops, soil, microbial consortia

Similarly to responses to biotic stresses, provoked by biological agents, like pathogens or insects (Verbon et al., 2017), plants respond to abiotic stresses, such as mineral nutrient deficiencies. Some of these responses are located at the effector site but others are systemic, inducing defense responses in the entire plant (Pieterse et al., 2014). Among the systemic responses is reported the Induced Systemic Resistance (ISR) (Romera et al., 2019). ISR is induced by beneficial rhizobacteria or by rhizofungi (Pii et al., 2016). The ways beneficial rhizosphere microorganisms elicit ISR is not totally understood but several substances produced by these microorganisms, like volatile organic compounds or siderophores that interact with the plants, have been proposed as elicitors (Martínez-Medina et al., 2017; Romera et al., 2019). Hormones and signaling molecules, either produced by the microorganisms or generated by the plants upon interaction with them, are also implicated in the ISR and mineral nutrient deficiency responses. Among them, jasmonic acid, ethylene, auxin and nitric oxide play a key role (Romera et al., 2019; Pescador et al., 2022). Some years ago, it was found that the *MYB72* gene, encoding a transcription factor (TF), was greatly induced in *Arabidopsis thaliana* roots upon treatment with *Pseudomonas simiae* (Van der Ent, 2008). *A. thaliana myb72* mutant plants can not develop ISR. This suggests that this TF plays a key role in the transduction pathway leading to ISR (Van der Ent, 2008; Zamioudis et al., 2015). Elucidating the main nodes of interconnection between the pathways regulating microbe-elicited ISR and mineral uptake is critical for optimizing the use of plant mutualistic microbes in agriculture. The Research Topic updates latest findings related to the roles of ISR eliciting microbes in crops.

The Research Topic was launched in late 2021 in the section “*Plant Symbiotic Interactions*” of *Frontiers in Plant Science* to continue further this thematic series (Lucena et al., 2021a). This second call includes 5 additional articles, three original research articles (Liu et al.; Qian et al.; Shan et al.) and two review papers (Qin et al.; Zhu et al.) by 34 authors.

Interplay between beneficial plant-microbe interactions and biotic and abiotic stress conditions has been observed and intensively studied (Romera et al., 2019; Usman et al., 2021). In fact, in addition to plant tolerance and plasticity for adaptation to harmful environements, beneficial miroorganisms will allow a kind of fortification of the plant physiology. The study by Liu et al. is dealing with an abiotic stress tightly linked to ongoing climate change, namely with heat stress. Authors, compared the effect of mild or high heat stress over several physiological indexes of *Rhododendron simsii* and on the posible changes of bacterial and fungal soil communities. The results showed that *R. simsii* may cooperate with soil microbial communities to obtain nutrients from the soil to help them resist heat stress when this stress is mild.

The role of the endophyte *Pseudomonas* sp. MCS15 through the production of a glucuronic acid and its interaction with ethylene inhibiting heavy metal uptake in rice was studied by Qian et al.. The Authors aimed at increasing evidence that endophytic bacteria can regulate plant hormone levels to help their hosts counteract adverse effects imposed by abiotic and biotic stresses. They showed that inoculation with MCS15 significantly inhibited the expression of ethylene biosynthetic genes and thus reduced the content of ethylene in rice roots. Using both precursors and inhibitors of ethylene biosynthesis, the author´s results revealed that the endophytic bacteria MCS15-secreted glucuronic acid inhibited the biosynthesis of ethylene and thus weakened iron uptake-related systems in rice roots, which contributed to preventing the Cd accumulation.

Abiotic stress is also connected to a lack of nutrients occuring in poor or affected environments. Implication of beneficial plant root associations with soil microbes becomes dissected at the molecular level revealing a symbiotic transportome. A broad interest has been developed for this topic during the last years (*e.g*. Garcia et al., 2016; Guerrero-Galán et al., 2018; Garcia et al., 2020; Lucena et al., 2021b). In this context, the study by Shan et al. dealing with the beneficial effects of mycorrhizal fungi for orchid growth is clearly demonstrating an improvement in nitrogen uptake. Previous studies had reported orchid growth promotion by such fungi. Here, the authors analyzed growth, transcriptomic and metabolic parameters of the medicinal important orchid *Dendrobium officinale* in interaction with the fungus *Mycena* sp., called MF23. Several plant genes involved in N transport and assimiation have been found to become upregulated upon symbiotic interaction.

In addition to mitigation of abiotic stress, beneficial interactions are playing also an important role in defense stimulation limiting pathogenic attacks (Pieterse et al., 2014). Better understanding of such interactions will surely become more important with respect to their applications in challenging agroecosystems. The review by Qin et al. aims at a systematic analysis of published data concerning the interplay between arbuscular mycorrhizal fungi (AMF) and pathogenic microbes from 36 studies including 650 observations. This meta-analysis revealed a tight link between AMF-stimulated plant growth and reduced pathogenic harm levels. AMF root length colonization was taken as best parameter for this analysis.

More generally, the review by Zhu et al. is related to the mechanisms underlying how plants recognize beneficial rhizobacteria which are generally called as plant growth-promoting rhizobacteria (PGPR). These PGPRs can be recognized as microbial associated molecular patterns (MAMPs), commonly called as pathogen associated molecular patterns (PAMPs), by diverse plant pattern recognition receptors (PRRs) further triggering host defense responses. The authors described that for establishing mutual benefits with the hosts, PGPRs have developed strategies to weaken the activation of host defense systems. Moreover, the process of the PGPR-induced ISR in plants can be regulated by root hair-specific syntaxins and non-coding RNAs. However, it remains elusive how plants balance between microbial recognition and defense activation. Moreover, the transferring mechanisms of small RNAs from roots to shoots for provoking ISR need to be deeply explored.

In conclusion, this second part of the Research Topic, bringing together 5 more articles dealing with plant-microbe associations in the context of plant defense and systemic resistence, reflects well the ongoing research in this area. In the light of agricultural demands to improve plant tolerance and growth, questions linked to this Research Topic remain challenging.

## Author contributions

CL and SDZ wrote the manuscript. All Editors contributed and approved this editorial.
